# The immunomodulatory function and antitumor effect of disulfiram: paving the way for novel cancer therapeutics

**DOI:** 10.1007/s12672-023-00729-9

**Published:** 2023-06-16

**Authors:** Sijia Zhang, Yan Zong, Leichong Chen, Qianwen Li, Zhenyu Li, Rui Meng

**Affiliations:** 1 Cancer Center, Union Hospital, Tongji Medical College, Huazhong University of Science and Technology, Wuhan, 430022 China; 2 Institute of Radiation Oncology, Union Hospital, Tongji Medical College, Huazhong University of Science and Technology, Wuhan, 430022 China

**Keywords:** Disulfiram (DSF), Tumors, Therapeutic drugs, Immune regulation, Programmed cell death-ligand 1 (PD-L1)

## Abstract

More than 60 years ago, disulfiram (DSF) was employed for the management of alcohol addiction. This promising cancer therapeutic agent inhibits proliferation, migration, and invasion of malignant tumor cells. Furthermore, divalent copper ions can enhance the antitumor effects of DSF. Molecular structure, pharmacokinetics, signaling pathways, mechanisms of action and current clinical results of DSF are summarized here. Additionally, our attention is directed towards the immunomodulatory properties of DSF and we explore novel administration methods that may address the limitations associated with antitumor treatments based on DSF. Despite the promising potential of these various delivery methods for utilizing DSF as an effective anticancer agent, further investigation is essential in order to extensively evaluate the safety and efficacy of these delivery systems.

## Introduction

As a promising antitumor drug, disulfiram (DSF) has brought new hope for its repurposing. DSF has been utilized for the treatment of alcohol dependence for more than 60 years, owing to its established and advantageous safety profile [1–3]. DSF is widely distributed in human tissues, with high concentrations in the kidney, pancreas, liver, and gastrointestinal tract [2]. DSF has antitumor activity against cancers including bladder cancer [4], intestinal cancer [5], acute and chronic lymphocytic/myeloid leukemia [6, 7], breast cancer [8], colorectal cancer [9], non-small cell lung cancer (NSCLC) [10], ovarian cancer [11], and kidney cancer [12]. An accumulating number of clinical trials are currently underway or have been completed to evaluate the antitumor efficacy of DSF. Clinical trials examining DSF’s efficacy in advanced solid tumors and newly diagnosed NSCLC have provided evidence supporting the notion that DSF holds potential as a promising candidate for cancer treatment (Table 1). Previous studies have focused on the direct tumoricidal function of diethyldithiocarbamate (DTC), a metabolite complex of DSF [13]. Despite extensive research on DSF's therapeutic applications, its impact on cellular immune response remains not well-understood. Thus, this review focuses on exploring potential mechanisms of immune cell modulation by DSF. And its immunotherapeutic antitumor effects, which in turn provide new references for broadening the antitumor function and mechanism of DSF.

**Table 1:** Clinical study of disulfiram in the treatment of solid tumors

Numbers	Trial ID	Initiation Date	Tumor type	Drug intervention	Phase	Number of recruits	Status	Country
1	NCT03363659	March 2018	Glioma	DSF, Copper gluconate, TMZ	II	40	Ongoing, No recruitment	The United States
2	NCT02678975	January 2017	Glioma	DSF, Cu^2+^, Alkylating agent	II, III	88	Recruited to complete	Norway, Sweden
3	NCT03151772	January 2018	Glioma	DSF, Metformin	I	3	Terminated	Swedish
4	NCT02715609	June 2016	Glioma	DSF, Copper gluconate, TMZ	I, II	36	Recruiting	The United States
5	NCT01907165	October 2013	Glioma	DSF, Copper gluconate, TMZ	I	21	Recruited to complete	The United States
6	NCT03034135	March 2017	Glioma	DSF, Copper gluconate, TMZ	II	23	Recruited to complete	The United States
7	NCT01777919	January 2017	Glioma	DSF, Cu^2+^, TMZ	II	32	-	Greece
8	NCT02770378	November 2016	Glioma	DSF, Other chemotherapeutic drugs	I, II	10	Recruited to complete	Germany
9	NCT02101008	March 2010	Melanoma	DSF, Chelating zinc	II	12	Recruited to complete	-
10	NCT00256230	January 2002	Melanoma	DSF	I, II	7	Recruited to complete	The United States
11	NCT00571116	September 2006	Melanoma	DSF, Arsenic trioxide	I	9	Recruited to complete	The United States
12	NCT03714555	October 2019	Pancreatic cancer	DSF, Copper gluconate, Other chemotherapeutic drugs	II	1	Recruited to complete	The United States
13	NCT02671890	February 2016	Pancreatic cancer	DSF, Gemcitabine	I	74	Recruiting	The United States
14	NCT04265274	January 2020	Breast cancer	DSF, Cu^2+^, Vinorelbine	II	28	Recruiting	Slovakia
15	NCT03323346	September 2017	Breast cancer	DSF, Cu^2+^	II	150	Recruiting	Czech
16	NCT01118741	May 2010	Prostate cancer	DSF	–	19	Recruited to complete	The United States
17	NCT02963051	July 2017	Prostate cancer	DSF, Cupric chloride, Copper gluconate	I	9	Terminated (Poor response)	The United States
18	NCT04521335	May 2021	Multiple myeloma	DSF, Copper gluconate	I	38	Recruiting	The United States
19	NCT03950830	May 2019	Germ cell neoplasm	DSF	II	20	Recruiting	Slovakia
20	NCT00312819	March 2006	Lung cancer	DSF, Other chemotherapeutic drugs	II/III	60	Recruited to complete	Israel
21	NCT00742911	July 2008	Tumor	DSF, Copper gluconate	I	21	Recruited to complete	The United States

*DSF* Disulfiram, *TMZ* Temozolomide

## Chemical structure of disulfiram

DSF is an off-white crystalline powder that exhibits poor solubility in aqueous media, but displays varying degrees of solubility in organic solvents, such as ethanol and ether [14]. In addition to possessing a small molecular weight of 296.54, DSF has a density of 1.30 and a melting point range of 70–72 °C [14]. The chemical formula of DSF is C_10_H_20_N_2_S_4_ [15]. Its antitumor activity was found to be Cu^2+^-dependent, with two sulfur atoms interacting with Cu^2+^ to form a copper-containing complex that demethylates DNA, enhances antigen-presenting cell (APC) activity and expression of the RARB-related proteins [15]. Thereby inhibiting the proliferation and growth of tumor cells. Safi, Rachid et al. [16] reported that DSF exhibited high cytotoxicity to tumor cells in the presence of Cu^2+^. In addition, complexes of DSF or its cellular metabolite diethyl dithiocarbamate with Cu^2+^ inhibit the proteasomal protein degradation system in cancer cells [17–19]. As the functional proteasome system is responsible for maintaining coordinated expression of cell cycle regulators and proteins involved in apoptosis control. DSF/Cu^2+^ complexes inhibit proteasome activity, leading to increased ubiquitination of proteins and the accumulation of cytotoxic proteins, which disrupt protein homeostasis in vivo and induce cancer cell death [20, 21].

## Pharmacology and metabolism of disulfiram

**Fig. 1:**
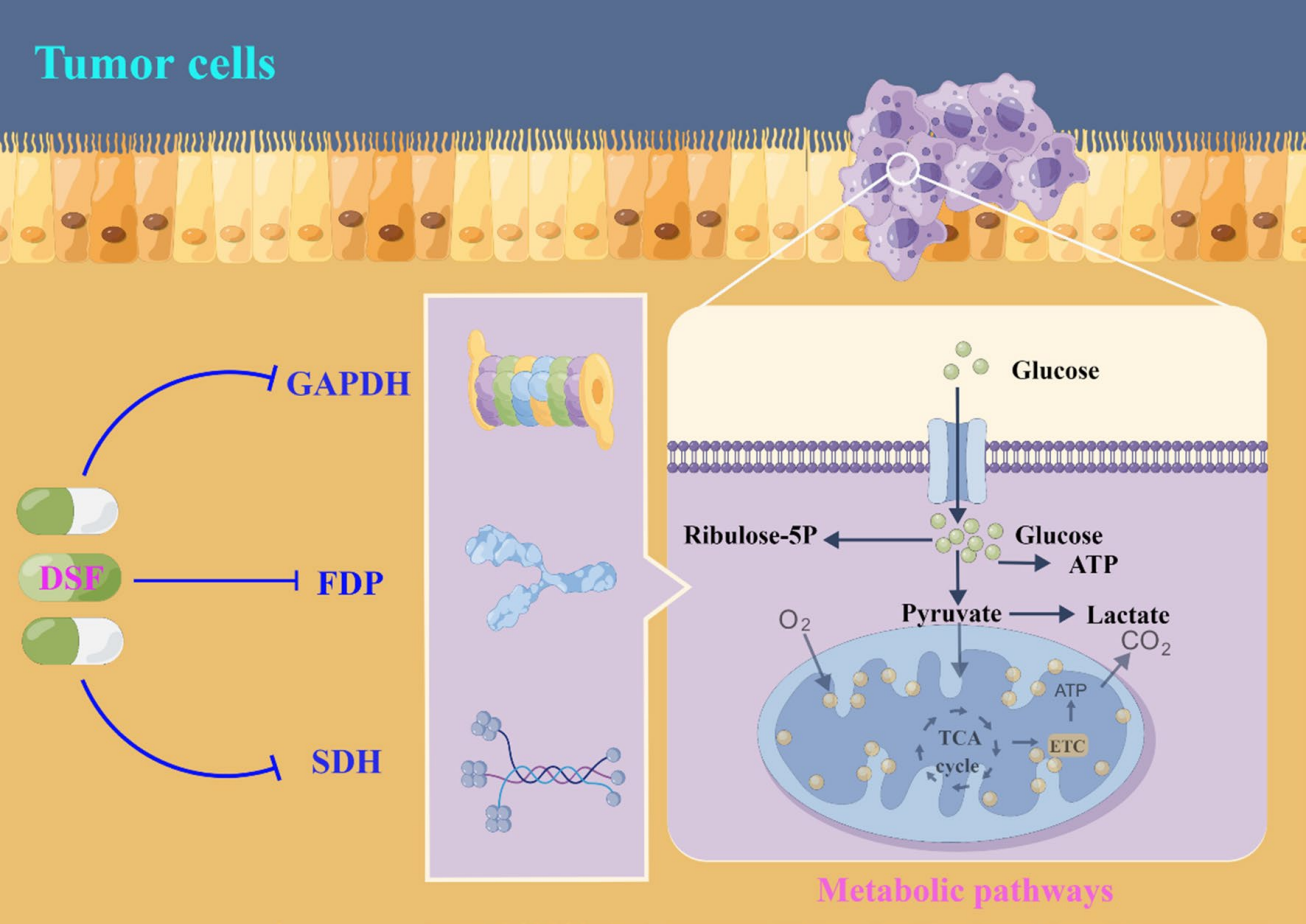
DSF can inhibit glyceraldehyde-3-phosphate dehydrogenase (GAPDH), fructose-l, 6-bisphosphate dehydrogenase (FDP), and succinate dehydrogenase (SDH), which are key enzymes involved in mitochondrial respiratory metabolism. By Figdraw (www.figdraw.com) and Adobe illustrator

**Fig. 2:**
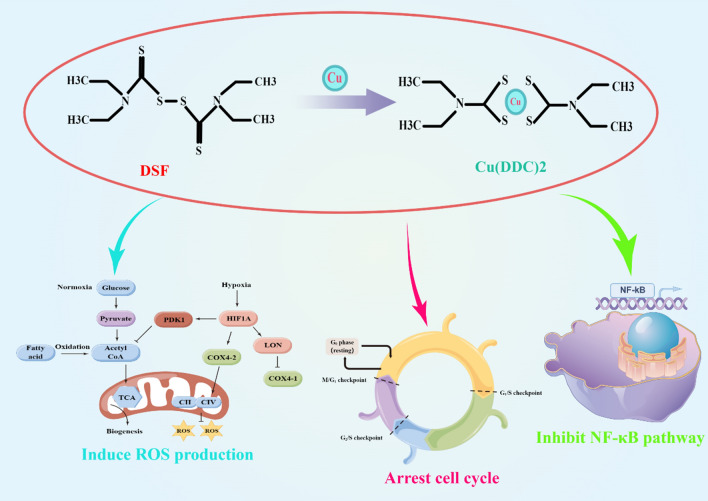
DSF combined with copper ions extensively affects tumor cell biological functions. By Figdraw (www.figdraw.com) and Adobe illustrator

DSF has bio-oxidative transformation properties, which can produce sulfate, methyl-mercaptan, and formaldehyde metabolites [22]. Metabolism of ethanol in the liver is predominantly executed by acetaldehyde dehydrogenase (ALDH) isoenzymes, with minor contributions from P450 monooxygenase and catalase [23]. Upon alcohol ingestion, ethanol is oxidized to produce acetaldehyde, which is further oxidized by ALDH to form acetate [22, 24]. Aside from its inhibition of ALDH, DSF can also impede other key enzymes involved in critical metabolic pathways, including glycolysis, the tricarboxylic acid cycle, and the pentose phosphate shunt [25–27]. DSF is capable of inhibiting crucial enzymes involved in mitochondrial respiratory metabolism, such as glyceraldehyde-3-phosphate dehydrogenase (GAPDH), fructose-l, 6-bisphosphate dehydrogenase (FDP), and succinate dehydrogenase (SDH) [28] (Fig. 1). Furthermore, DSF functions as an inhibitor of dopamine-β-hydroxylase (DBH), an enzyme responsible for catalyzing the conversion of dopamine into norepinephrine during catecholamine synthesis [29]. DSF is metabolized mainly in the liver, where disulfide bonds are broken to produce two molecules of diethyldithiocarbamate (DDC) [30]. Acidic environments cause DDC to form a complex with Cu^2+^ resulting in Cu (DDC)2 [31, 32]. Cu (DDC)2 is more stable than DDC and has its own antitumor activity. Importantly, the concentration of Cu (DDC)2 is significantly higher in tumor tissues compared to other tissues (liver and brain tissues, etc.) and blood, showing specific antitumor advantages and potential [33] (Fig. 2).

## Signaling pathways regulated by disulfiram

### Elevated production of reactive oxygen species (ROS) and suppression of the ROS/NF-κB signaling pathway

**Fig. 3:**
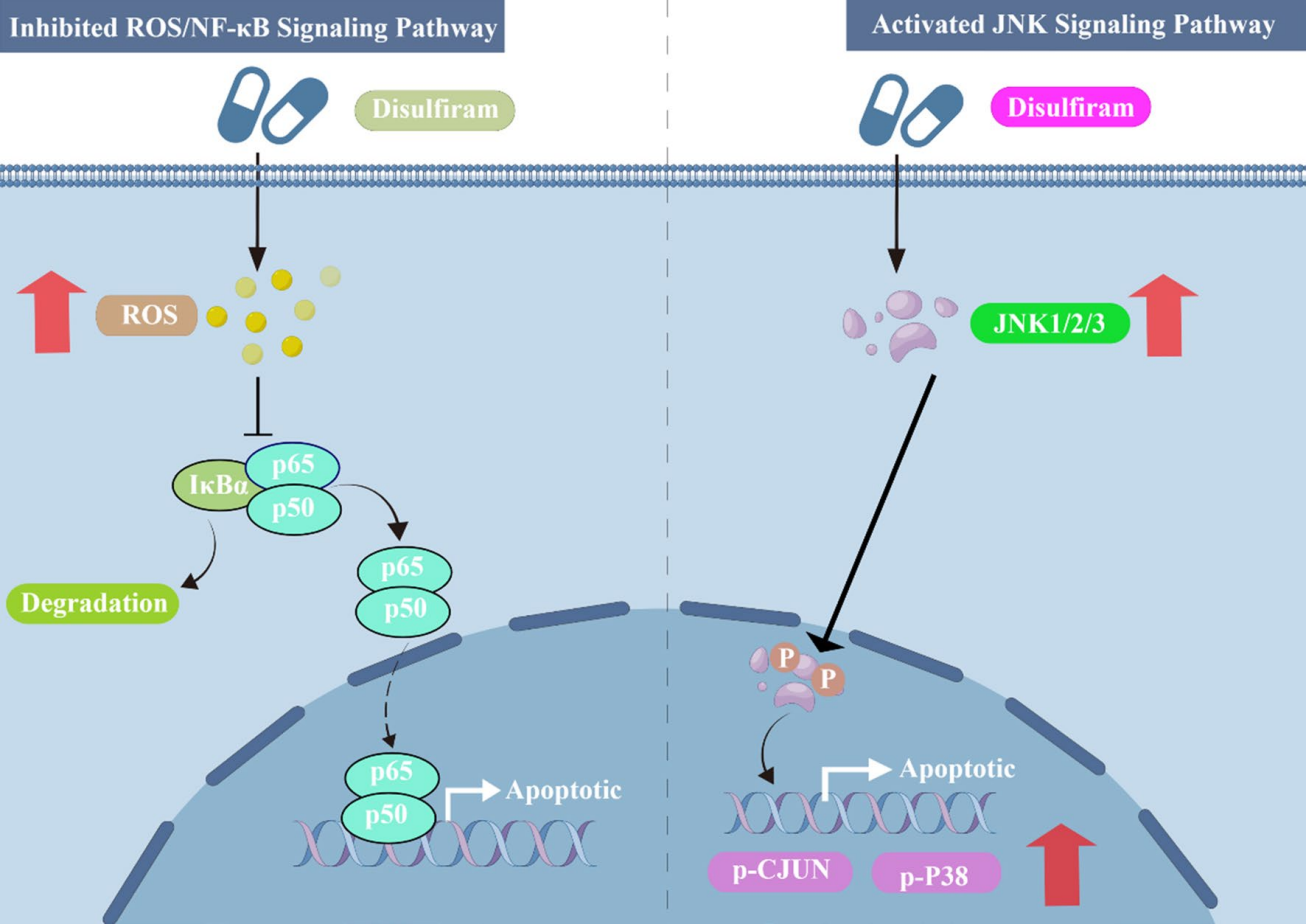
DSF is a strong inducer of ROS production and an effective proteasome inhibitor, resulting in the inhibition of NF-κB **(Left)**. DSF could inhibit tumor cell growth by activating the JNK pathway and intracellular levels of phospho-JNK, phospho-CJUN and phospho-P38 were increased after DSF treatment **(Right)**. By Figdraw (www.figdraw.com) and Adobe illustrator

Research has shown that cancer cells generally exhibit higher levels of ROS than normal cells, and various studies have confirmed that DSF is capable of instigating ROS production in numerous types of cancer cells [34]. Which further increases ROS in tumor cells, thereby disrupting the oxidative homeostasis and inducing apoptosis [35, 36]. Moreover, DSF has been shown to effectively inhibit NF-κB pathway activity and augment the apoptotic impact of 5-fluorouracil (5-FU) on colorectal cancer cells when administered in conjunction with 5-FU [37, 38]. Furthermore, in breast cancer cells and human glioblastoma cell lines, DSF was observed to reverse chemoresistance by inhibiting NF-κB [39, 40]. Likewise, studies have demonstrated that DSF/Cu^2+^ complexes can effectively suppress NF-κB pathway activity and reverse chemoresistance to the antitumor drug gemcitabine in both colon and breast cancer cell lines [41]. In summary, DSF is a potent inducer of ROS generation and a highly effective proteasome inhibitor, ultimately leading to suppression of the NF-κB pathway (Fig. 3).

### Activate the JNK signaling pathway

The c-Jun N-terminal kinase (JNK) signaling pathway plays a pivotal role in regulating apoptosis by modulating the expression of apoptotic proteins as well as the activity of Bcl-2 family proteins. In studies with breast cancer [42], head and neck squamous cell carcinoma [35, 36], acute myeloid leukemia [43] and myeloma cells [44], Studies have revealed that DSF impedes tumor cell proliferation by activating the JNK pathway, thereby elevating the intracellular levels of phosphorylated JNK, phosphorylated c-Jun, and phosphorylated P38 upon DSF exposure (Fig. 3).

### Inhibition of the NPL4/p97 signaling pathway induces antitumor effects

NPL4, a molecular target of DSF action for tumor suppression and a bridging factor for the p97 isolase [2]. It is a key link in a variety of protein and stress response pathways. Particularly, as cancer cells metabolize at a different rate to normal cells. It becomes more dependent on the NPL4/p97 pathway for the degradation of remaining waste proteins [45]. Skrott, Zdenek et al. [46] found that the DSF/Cu^2+^ targets the p97-NPL4-UFDI pathway upstream of the proteasome. DSF functions by binding to NPL4, which causes aggregation of NPL4 and subsequent inactivation of the P97 isolase, ultimately leading to accumulation of misfolded proteins within cells and ultimately resulting in cell death [46]. A recent study conducted by Chen, Cunte et al. [45] reported that DSF was capable of triggering apoptosis and impeding the proliferation of malignant T cells through engagement with the NPL4-dependent ubiquitin–proteasome pathway. These findings highlight the potential utility of using DSF as a therapeutic approach for treating T cell malignancies. Skrott, Zdenek et al. [47] made an intriguing discovery, demonstrating that DSF's anticancer properties are largely due to its targeting of NPL4 rather than its inhibition of ALDH.

### Blocking cell cycle-related signaling pathways

Many anticancer therapies function by impeding the cell cycle, and studies have proven that DSF/Cu^2+^ complexes are capable of invoking notable growth inhibition and apoptosis in tumor cells through the disruption of normal cell cycle progression [43]. In acute myeloid leukemia, DSF/Cu^2+^ enhances the expression of the oncogene FOXO and inhibits the expression of the oncogene MYC, inducing G0/G1 cell cycle arrest and tumor cell apoptosis [43]. DSF/Cu^2+^ also induced G2/M cell cycle arrest and apoptosis in multiple myeloma cells [44] and nasopharyngeal carcinoma cells [36].

### Directly binding to the FROUNT and interfering with FROUNT/chemokine/receptor signaling pathway

Tumor microenvironment-associated macrophages influence tumor progression and immunotherapeutic efficacy [48]. DSF binds directly to FROUNT and interferes with FROUNT/chemokine/receptor interactions, reducing macrophage protumor activity and slowing tumor progression [49]. Combined DSF and programmed cell death protein 1 (PD-1) antibody treatment markedly increased the number of granzyme B (GZMB)-positive CD8^+^ T cells in the tumor [49]. Thus, FROUNT inhibitor, DSF, is able to enhance the response to immune checkpoint therapy by modulating macrophages.

### Inducing immunogenic cell death signaling pathway

Immunogenic cell death (ICD) can enhance the antitumor immune response and effectively improve the effect of antitumor immunotherapy [50]. In colorectal cancer cells, DSF induced apoptosis and increased the expression of ICD signaling molecules, calreticulin and heat shock protein 70 [51]. Therefore, this mechanism suggests that DSF may be involved in antitumor effects by modulating the immune system in addition to the direct action of the drug.

## Advantages and obstacles in the wide use of disulfiram

DSF offers numerous advantages compared to conventional antitumor therapies. Firstly, it has negligible side effects, as evidenced by its use as an anti-alcoholism drug for over 60 years with minimal adverse effects in the clinic [52]. Preclinical studies further demonstrate that DSF targeting cancer cells does not alter the body weight of mice, indicating its safety. Secondly, DSF/Cu^2+^ effectively kills therapy-resistant cancer cells, including cancer stem cells, thereby preventing tumor recurrence and metastasis [52]. Moreover, DSF/Cu^2+^ enhances the efficacy of conventional chemotherapy and chemoradiation, while remaining cost-effective [53].

However, there are challenges in implementing DSF in clinical use. There are several challenges associated with DSF’s clinical use, including the unknown active metabolites of DSF in vivo, inadequate assays to measure active derivatives in tumors, limited biomarkers to monitor its effects on both normal and tumor tissues, and insufficient knowledge regarding its selective toxicity towards cancer cells relative to healthy tissues. Further, when DSF is combined with other chemotherapeutic agents, there may be potential non-negligible side effects, such as increased gastrointestinal toxicity and ototoxicity [53]. Unexpected results in clinical trials underscore the importance of factoring in the toxicities and clinical benefits of DSF, along with the patient population to which it is applied, for its successful repurposing as an antitumor agent. Nevertheless, the prospects of using DSF as a cancer treatment are excellent, and future research aimed at exploring its molecular mechanisms and the development of clinical trials are needed to bridge the gap between basic research and clinical implementation.

## Disulfiram regulates immune cell function

### The role of DSF in the adaptive immune system

**Fig. 4:**
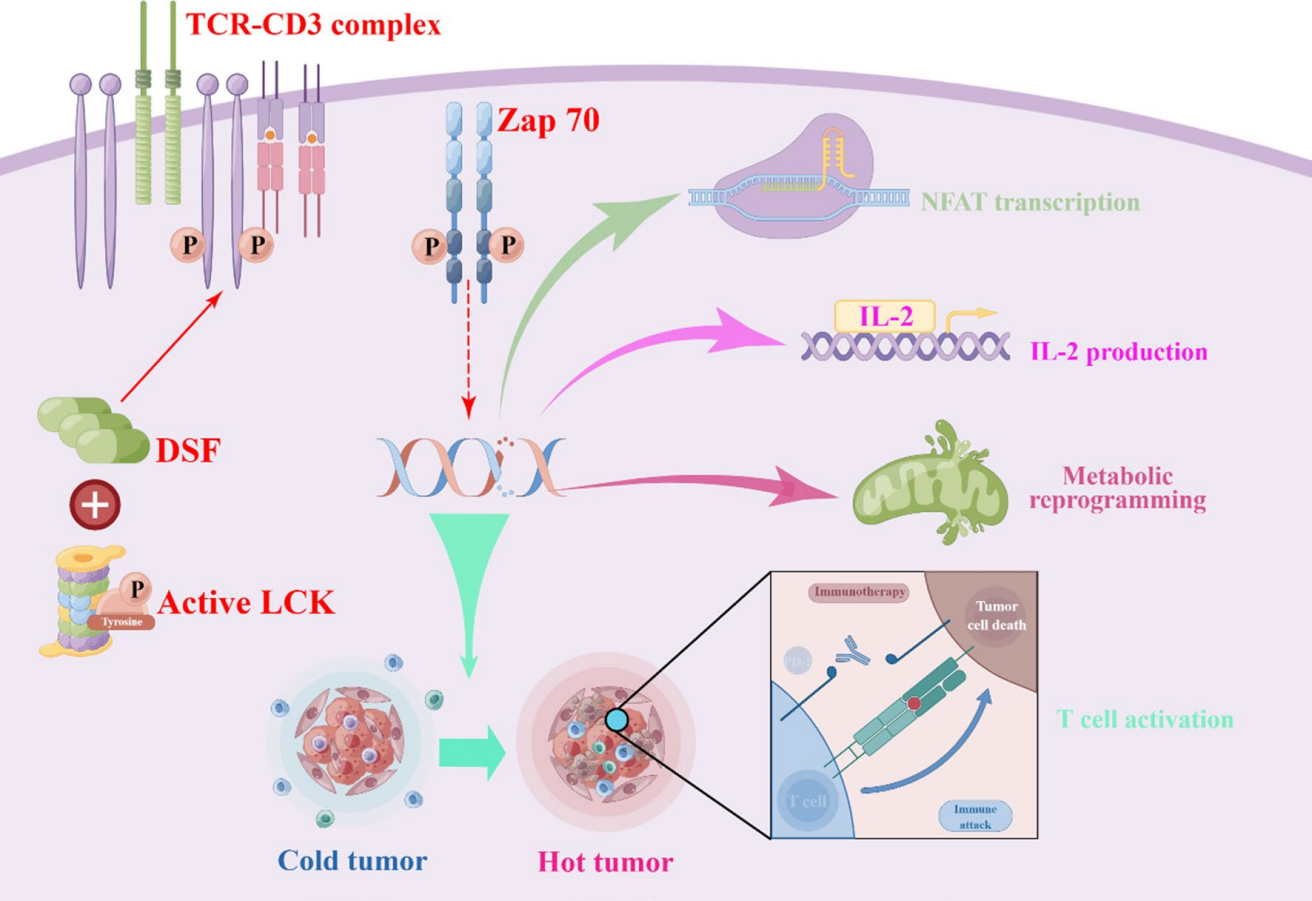
DSF covalently binds to active lymphocyte-specific protein tyrosine kinase (LCK) and enhances its tyrosine 394 phosphorylation, thereby promoting LCK kinase activity and boosting effector T cell function, interleukin-2 (IL-2) production, metabolic reprogramming, and proliferation. Furthermore, DSF promotes antitumor immunity by activating CD8^+^ T cells, and this effect was enhanced by anti-PD-1 co-treatment. By Figdraw (www.figdraw.com) and Adobe illustrator

The adaptive immune system is comprised of specialized cells and intricate processes that function in a highly precise and systemic manner to eradicate pathogens or curb their proliferation [54]. During the adaptive immune response, CD8^+^ T cells are the main type of lymphocytes and play an important role between tumor immunity and autoimmunity [55]. The recognition of tumor antigens by the TCR-CD3 complex must be correctly translated into the signaling events necessary to induce an effective immune response [56]. However, the tumor microenvironment inhibits the activation of CD8^+^ T cells to promote immune escape from tumors [57]. Therefore, effective promotion of CD8^+^ T cell antitumor responses is urgently needed. It was found that DSF directly binds to LCK via Cys20/23 and promotes its kinase activity, thereby enhancing the T cell effector response and enhancing the antitumor immunity of CD8^+^ T cells [58]. LCK belongs to the SRC kinase family and is the first molecule to be recruited into the TCR complex, either free or associated with CD8 or CD4 co-receptors [59]. Free LCK, associated with the TCR-CD3 complex, phosphorylates CD3, which activates the TCR signaling pathway [49] (Fig. 4). Whereas Wang, Qinlan et al. [60] found that DSF directly enhances T cell-mediated cytotoxicity and its antitumor function is eliminated when CD8^+^ T cells are depleted. Supporting that the antitumor function of DSF is dependent on the direct activation of CD8^+^ T cells, treatment with DSF/Cu^2+^ alone was observed to inhibit and upregulate PD-L1 expression by PARP1 and CD8^+^ T cells ratio was reduced. Moreover, the use of DSF/Cu^2+^ in conjunction with an anti-PD-1 antibody which jointly inhibits both PARP1 and the PD-1/PD-L1 pathway, not only resulted in reduced tumor cell viability but also led to an increase in the number of T cells that infiltrate the tumor. The combination of PARP inhibitor with anti-PD-1 antibody produced potent antitumor effects [60].

### DSF regulates cytokine release

Cytokines are a group of small molecule proteins with a wide range of biological activities [61]. They play a range of roles in tumourigenesis and development, regulating cell growth, differentiation and maturation, maintenance of function, immune response, and participating in inflammatory responses, wound healing and tumor regression by binding to the appropriate receptors [62]. Recent studies have shown that DSF can regulate cytokine release. Superoxide dismutase-1 (SOD1) is a critical factor in regulating redox-sensitive inflammatory signaling pathways in microglia. Over-expression of this gene could reduce ROS production and neurotoxic inflammation [63]. DSF can reverse the release of cytokines (including IL-6 and TNF-α) produced by SOD1 by inhibiting SOD1 expression [63]. In CD44^+^ T cells latently infected with HIV-1, DSF induces gene expression of HIV-1 without causing T cell activation [64]. Although it is not clear whether DSF can reactivate latent HIV-1 in vivo, DSF alone or in combination with other drugs may provide ideas for future therapeutic strategies. Franchi, S et al. [65] examined the effects of DSF, naltrexone and gamma-hydroxybutyric acid on the immune response in alcoholics and showed increased concentrations of IL-1, IL-4, IL-2 and IFN in the DSF group. In glomerulonephritis, DSF has been shown to attenuate macrophage activation by downregulating the expression of inflammatory cytokines and chemokines, such as TNF-α, CCL2, and CXCL9, as well as by decreasing the expression of CD86 and MHC class II in monocytes and macrophages [66]. The studies conducted thus far demonstrate that DSF is capable of modulating the secretion and release of cytokines. Further investigation into DSF's immunomodulatory properties in vitro may help to elucidate its precise antitumor mechanism, and potentially lead to the development of novel therapeutic strategies for treating various types of cancers.

### Up-regulation of immunogenic cell death by disulfiram

ICD refers to the process by which a drug induces the transition of cancer cells from a non-immunogenic state to an immunogenic state, thereby eliciting an antitumor immune response in vivo [67]. Tumor cells that have undergone immunogenic death release a diverse array of cytokines on their surface and express various signaling molecules, thereby triggering immune cells to identify and eliminate tumor cells within their microenvironment [68]. For example, by recruiting dendritic cells (DCs) to the tumor lesion and promoting their immune function [69]. At the same time, it produces a tumor-specific T cells response by recruiting and activating APCs to eliminate tumor cells [69]. The results show that DSF can act as an ICD inducer by up-regulating the expression of calreticulin (CRT) protein, an ICD-related molecule located on the cell membrane surface [51]. Because CRT plays a very important role in mediating ICD in tumor cells. It is an intramembrane protein highly expressed on the cell membrane surface, it can be recognized as a cytotoxic marker by the APC [70]. The increased level of CRT protein in cells after DSF treatment means that it can recruit more specific T cells to attack tumor cells and act using immunogenic antitumor effects.

### Disulfiram and immune checkpoint receptor

**Fig. 5:**
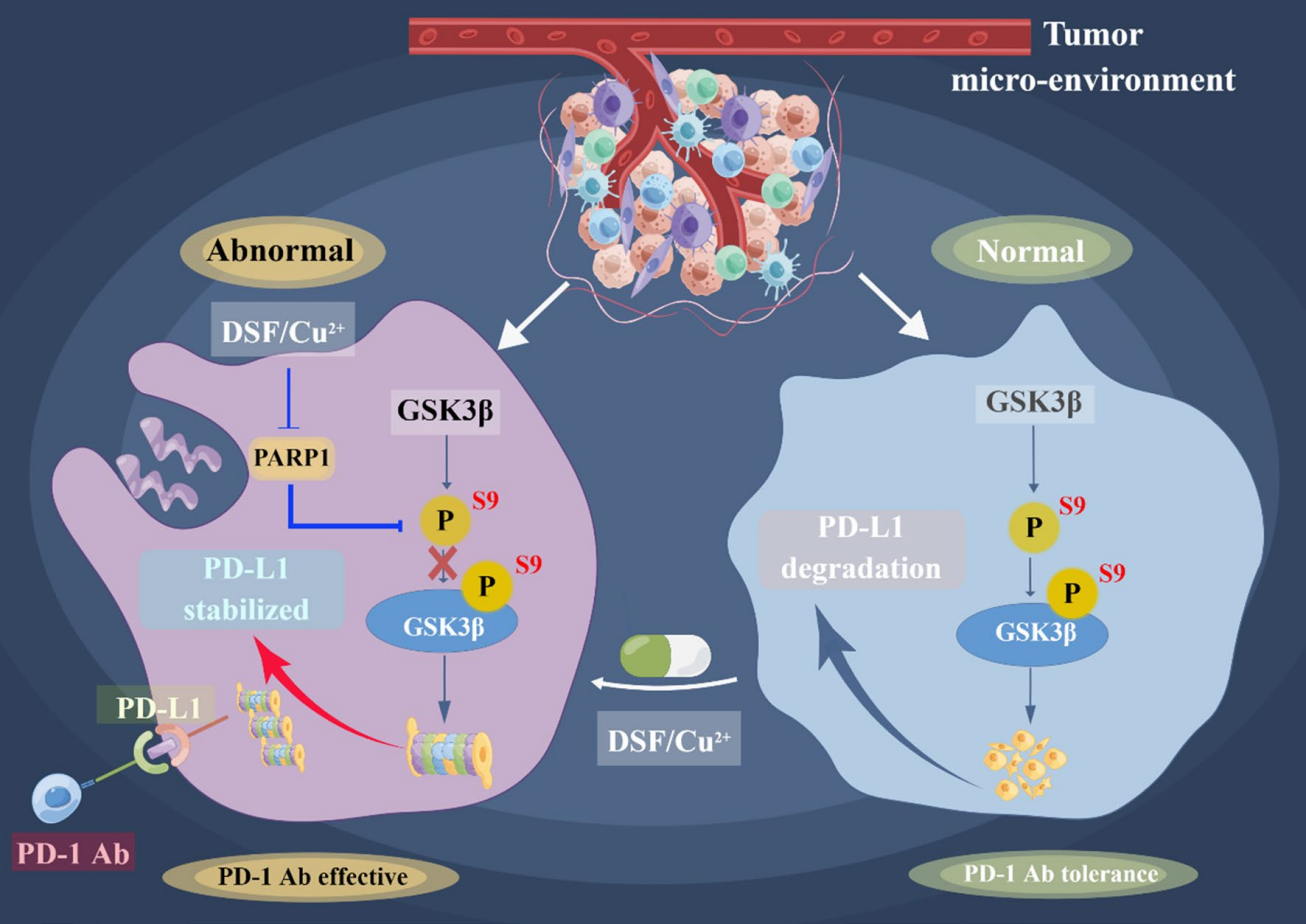
DSF/Cu^2+^ inhibited the activity of PARP1 and induced the phosphorylation of GSK3β at Ser9, thus inactivating GSK3β to reduce the degradation of PD-L1, which led to the suppression of antitumor immunity. The combination of DSF/Cu^2+^ and an anti-PD-1 monoclonal antibody may boost antitumor immunity efficacy. By Figdraw (www.figdraw.com) and Adobe illustrator

Immune checkpoint blockade (ICB), particularly targeting PD-1 and PD-L1, has demonstrated significant clinical value for patients afflicted with a range of cancers [71]. Studies have shown that DSF can upregulate PD-L1 expression by promoting DNMT1-mediated hypomethylation of IRF7 [72]. In a mouse model of 4T1 breast cancer, DSF was found to markedly enhance the efficacy of anti-PD-1 antibody treatment [72]. Analysis of tumor tissue samples from the combination treatment group revealed a significant increase in the presence of GZMB^+^ and CD8^+^ T cells [72]. The use of DSF in combination with anti-PD-1 antibodies has been demonstrated to yield significantly improved antitumor effects compared to using either treatment alone [72]. Additionally, DSF/Cu^2+^ has been shown to impede GSK3β activity via PARP1 inhibition, thereby leading to an upregulation of PD-L1 expression [73] (Fig. 5). While numerous studies have reported the promising synergistic antitumor effects of DSF in combination with other agents, additional research is necessary to fully evaluate its potential for use in immunotherapy. It is believed that as the research on the efficacy, mechanism and clinical aspects of DSF continues, a reasonable and effective combination regimen will be explored (Table 2).

**Table 2:** Disulfiram regulates immune cell function

Immune cell	Signal pathway	Function
CD8^+^ T cells	LCK-mediated TCR signaling	(1) DSF bolsters CD8^+^ T cells antitumor immunity through direct activation of LCK-mediated TCR signaling (2) DSF/Cu^2+^ in combination with anti-PD-1 antibody activated tumor-infiltrating CD8^+^ T cell
Macrophages	FROUNT/Chemokine/Receptor interactions	(1) Targeting FROUNT with DSF suppresses macrophage accumulation and its tumor-promoting properties (2) Reprogramed macrophage by secreting antitumor cytokines (e.g., TNF-α and IFN-γ) and reducing the level of immune suppressor B7-H4
Granzyme B^+^ cells	–	(1) DSF/Cu^2+^ in combination with anti-PD-1 antibody activated the expression of granzyme B
Cytokines	The SOD1 expression	(1) DSF can reverse the release of IL-6 and TNF-α produced by inhibiting SOD1 expression (2) DSF could increase the concentrations of IL-1, IL-4, IL-2 and IFN (3) DSF suppressed macrophage activation by reduced expression of TNF-α, CCL2, and CXCL9
Immunogenic cells	(1) The CRT expression (2) Calreticulin (3) Heat shock protein 70	(1) Recruiting more specific T cells to attack tumor cells and act using immunogenic antitumor effects
PD-L1	(1) DNMT1/IRF7 axis, (2) PARP1/GSK3β axis	(1) DSF enhanced PD-L1 expression via DNMT1-mediated IRF7 hypomethylation (2) DSF/Cu^2+^ could restrain GSK3β activity by inhibiting PARP1, leading to the upregulation of PD-L1 expression

## Research progress of antitumor effect of disulfiram

The antitumor potential of DSF has been demonstrated across numerous cancer cell models, with its activity being strongly linked to the formation of Cu (DDC)2. DSF/Cu (DDC)2 has been found to exhibit cytotoxicity against cancer cells, and is effective in reversing both acquired and hypoxia-induced antitumor drug resistance. Furthermore, DSF/Cu (DDC)2 has been shown to promote antitumor drug-induced apoptosis in various types of cancers including colon, breast, lung, liver, and brain tumors [74]. The current antitumor mechanisms of DSF includes the following.

### Disulfiram-induced cell death

DSF’s antitumor mechanism is copper-dependent and involves redox reactions. Cancer cells are known to contain a high number of copper transporters, which enables DSF to selectively target these cells by forming a complex with copper that facilitates its entry into cancer cells. This selective targeting allows DSF to specifically impede cancer cell survival without affecting healthy cells, which typically exhibit low levels of copper expression [39]. The interaction between DSF and Cu^2+^ generates extracellular ROS that prompt the initiation of apoptosis in cancer cells [75]. Furthermore, it has been demonstrated that DSF/Cu (DDC)2 can accumulate within cancer cells and conversely, trigger ROS production, ultimately culminating in apoptosis [12, 76].

### Inhibition of proteasomes

The functional activity of proteasomes is crucial in maintaining simultaneous expression of both apoptosis control proteins and cell cycle regulatory proteins, and thus, proteasome-mediated protein degradation plays a significant role in maintaining cellular homeostasis. Cancer cells are generally more vulnerable to abnormal proteasome inhibition than normal cells. In several cancer types, the combination of DSF with Cu^2+^ has been found to be a potent inhibitor of functional proteasomes [12]. Of particular interest, DSF/Cu^2+^ has been shown to effectively inhibit proteasome activity in breast cancer cells while having no effect on proteasome function in normal breast cells. This inhibition of proteasome activity by DSF/Cu^2+^ is thought to trigger the accumulation of poly-ubiquitinated proteins, and lead to cytotoxic protein aggregation of key proteins such as IκB, p27, Kip1 and c-Myc [77]. This accumulation of protein aggregates that is induced by DSF/Cu^2+^ mediated inhibition of proteasome activity is believed to disrupt normal cell cycle progression, resulting in apoptosis.

### Inhibition of cancer stem cells

Cancer stem cells (CSCs), which are also referred to as tumor stem cells, are a subpopulation of cells found within tumors that have the inherent capability to self-renew and produce diverse types of tumor cells [78]. CSCs are known to exhibit high rates of resistance to many standard chemotherapy treatments, rendering them a significant contributor to the recurrence of cancers. Promisingly, studies have demonstrated that DSF can enhance the cytotoxic effects of cisplatin by inhibiting the transcription factors responsible for stem cell function in several breast cancer cell lines [79]. In addition to the above-mentioned study, Guo, Fang et al. [11] have reported that DSF holds considerable potential for enhancing the cytotoxic effects of cisplatin in ovarian cisplatin-resistant ALDH-positive cancer-stem-like cells. This was achieved by inhibiting ALDH activity and inducing apoptosis, thereby sensitizing these cells to cisplatin treatment. Notably, DSF has also been shown to effectively reverse cisplatin resistance in testicular germ cell tumors by inhibiting ALDH activity [80]. It has been reported that the DSF/Cu^2+^ complex can be effective in eliminating multiple myeloma stem cells by inhibiting ALDH activity and suppressing stem cell-associated transcription factors such as Nanog and Oct4. In comparison to DSF alone, the DSF/Cu^2+^ complex demonstrated significantly greater efficacy in this regard [81].

### A novel approach to immunosuppressive therapy

Strongly immunosuppressive microenvironment is a major obstacle in tumor therapy. There is increasing evidence that DSF exerts antitumor activity through immune pathways. FROUNT, a chemokine signaling regulator, is highly expressed in macrophages, and its myeloid-specific deletion has been found to impair tumor growth. Studies have demonstrated that DSF can effectively inhibit FROUNT by directly binding to specific sites within the chemokine receptor binding domain of FROUNT, thereby obstructing interactions between FROUNT and chemokine receptors [49]. By inhibiting FROUNT and subsequently hindering macrophage responses, DSF can reduce the tumor-promoting activity of macrophages, thus exhibiting potent antitumor activity [49].

The NOD-, LRR-, and pyrin domain-containing protein 3 (NLRP3) inflammasome serves a pivotal role in mediating the innate immune system. Deng, Wenmin et al. [82] found that DSF effectively inhibited NLRP3 inflammatory vesicle activation and suppressed cellular scorching. Additionally, DSF has been demonstrated to inhibit the release of lysosomal histone protease B into the cytoplasm, a process which in turn leads to the inactivation of NLRP3 inflammatory vesicles [82]. The aforementioned mechanism via the inhibition of lysosomal histone protease B release and inactivation of NLRP3 inflammatory vesicles, has been shown to engender profound therapeutic effects in LPS-induced peritoneal inflammation and MSU-induced gout inflammation following DSF treatment [82].

Another study has shown that DSF/Cu^2+^ complexes can upregulate PD-L1 expression, ultimately inhibiting T-cell infiltration by modulating PARP1 activity and increasing GSK3β phosphorylation at Ser9. Notably, when used in combination with anti-PD-1 antibody therapy, DSF/Cu^2+^ has been demonstrated to exhibit superior antitumor efficacy to either agent alone [73].

The development of new drugs is a costly and time-consuming process. Repurposing drugs that are already in clinical use as potential treatments for cancer represents an appealing alternative, as it can expedite the process of drug development and lower costs. In a recent study, Ou, An-Te et al. [83] reported the development of a DSF-LF nanoparticulate system (DSF-LF NP) for synergistically combining the immunosuppressive activities of both DSF and lactoferrin (LF). The DSF-LF NPs demonstrated an ability to effectively block pyroptosis and suppress the release of inflammatory cytokines from macrophages when tested in cell culture systems [83]. In animal studies, treatment with DSF-LF NPs was found to generate significant therapeutic effects on lipopolysaccharide (LPS)-induced sepsis, murine colitis, and ulcerative colitis (UC) [83]. Disulfiram-loaded lactoferrin nanoparticles are presently being extensively investigated in advanced solid tumor studies, emerging as a promising therapeutic approach with significant potential in cancer research.

Zhao, Pengfei et al. [84] have reported on an albumin-based biomimetic delivery system for the co-delivery of DSF/Cu^2+^ and the macrophage modulator regorafenib (Rego), utilizing a “two-birds-one-stone” strategy for dual targeting of both glioma cells and TAM2. Their system was found to reprogram macrophages by enhancing the secretion of antitumor cytokines (such as TNF-α and IFN-γ) and reducing the levels of the immune suppressor B7-H4 [84]. Significantly, this system also influenced T cells by inhibiting Treg cells and activating CD8^+^ T cells, ultimately eliciting strong cellular immunity against glioma cells [84].

## Delivery systems for DSF

DSF's oral dosage form is ineffective for cancer treatment due to its instability in the gastric environment and rapid degradation in the body [85], necessitating the development of a more efficient drug delivery system. DSF formulations can prevent degradation, improve circulation half-life, and promote enhanced accumulation and release in tumor tissues while minimizing exposure in normal tissues. Various DSF delivery strategies have been developed, including physical encapsulation and conjugation methods. Physical encapsulation uses liposomes or nanoparticles to protect the drug from degradation and improve its half-life in circulation. These systems can also target tumors through the enhanced permeability and retention effect, increasing drug accumulation in the tumor site. Conjugation methods bind DSF to other molecules, such as antibody fragments or peptides, to target specific cancer cell receptors, boosting drug uptake and reducing exposure in normal tissues to achieve better efficacy and reduce side effects. Development of more efficient drug delivery systems for DSF is essential to enhance stability, increase tumor tissue accumulation and release, and decrease exposure in normal tissues, leading to improved therapeutic outcomes.

### Polymer nanoparticles

Researchers have utilized poly lactic-co-glycolic acid (PLGA), an FDA-approved biodegradable polymer, in multiple studies to prepare nanoparticle (NP) formulations of DSF [86]. McConville et al. used PLGA mini-rods, prepared through a “hot melt extrusion” method, to treat glioblastoma multiforme via stereotactic injection into the brain [86]. Wang et al. prepared DSF PLGA NPs using an emulsion-solvent evaporation method to treat liver cancer, which improved DSF stability and prolonged its half-life in vivo from 2 min to 7 h [87, 88]. The properties of DSF PLGA NPs were influenced by the selection of the PLGA polymer, stabilizer, and sonication time [88]. DSF encapsulated PEG-PLGA NPs have been shown to improve tumor site delivery and prolong systemic circulation half-life. Fasehee et al. attached folate to the surface of DSF PEG-PLGA NPs, increasing cellular uptake in tumor cells expressing folate receptors [89]. Another study developed a PEG-PLA/poly(ε-caprolactone) hybrid NP for DSF delivery and increased loading capacity via optimization of PEG-PLA/PCL content ratios [89].

### Lipid NPs

Lipid nanoparticles (NPs) have shown potential as DSF delivery systems in various studies. Banerjee et al. used vitamin E-TPGS surface-modified lipid NPs with high drug encapsulation efficiency to demonstrate better antitumor efficacy than free or unmodified DSF [90]. Zhang et al. designed pH-responsive TAT peptide-decorated lipid NPs for DSF delivery, with TAT peptide modified with pH-responsive PEG-PGA to prevent non-specific cellular uptake in normal tissues and enhance intra-tumor penetration [91]. Liu et al. used biotin-PEG-DSPE to modify DSF-lipid NPs, enhancing tumor targeting via biotin receptors in cancer cells. In vivo studies showed that biotin-PEG-DSPE modified DSF lipid NPs effectively inhibited breast cancer growth in mice tumor models [92].

### Micelles

Micelles have also been studied as delivery systems for DSF, with the drug loaded in the hydrophobic region of the micelles [93–95]. To improve micelle stability, Duan et al. prepared cross-linked micelles using redox-sensitive bonds, releasing DSF under tumor redox conditions [96]. Miao et al. developed mixed micelles consisting of PEG-PLGA, PCL, and MCT, improving drug loading capacity and stability [97]. Combination therapy was explored with DOX and DSF co-delivered using core–shell micelle NPs composed of poly(caprolactone)-b-poly(L-glutamic acid)-g-methoxy poly(ethylene glycol) or micelle NPs formed by a polymer-DOX conjugate. These systems can release both drugs in response to the acidic tumor environment, displaying potential for treating drug-resistant cancers and enhancing drug delivery into tumors [98].

### Nanocrystals

Nanocrystals have emerged as a novel delivery system for poorly water-soluble drugs, consisting of pure drug crystals without or with minimal excipients. Recent studies have developed hybrid DSF-PTX nanocrystals for co-delivery of these two drugs [99, 100]. DSF-PTX nanocrystals were prepared using an anti-solvent precipitation method and stabilized with β-lactoglobulin [96, 97]. The optimized formulation comprised rod-like nanoparticles with high drug loading efficiencies, displaying significantly better cellular uptake and effectively killing PTX-resistant lung cancer cells [96, 97]. This system also demonstrated better efficacy than paclitaxel alone in inhibiting tumor growth in an in vivo MDR lung tumor model.

### DSF conjugates

Drug conjugates refer to prodrugs where drugs are chemically linked with small molecules, polymers (such as PEG), lipids (such as cholesterol), or biomacromolecules (such as albumin) [101, 102]. These conjugates have superior physicochemical properties, minimal premature drug release or leakage, and are a promising approach for enhancing drug delivery [103]. Several studies have prepared conjugate delivery systems of DDC, a metabolite of DSF. He et al. used lactobionic acid-modified PDA-PEG-LBA polymer to prepare DDC conjugates as a tumor-targeting delivery carrier, showing potent antitumor activities in a peritoneal metastatic ovarian tumor model [104]. Bakthavatsalam et al. synthesized a prodrug containing a GGT-responsive linker [105], while Pan et al. synthesized an H2O2-responsive DDC prodrug, both effectively inducing cancer cell death [106]. Lipid-conjugated DSF prodrugs have the potential to be used for cancer therapy, while DSF polymer prodrugs synthesized through RAFT polymerization could effectively induce apoptosis in melanoma cells without significant toxicity to normal cells [107, 108].

## Future perspectives

The development of DSF-based cancer therapy offers great potential as a potent antitumor agent. However, the formulation used greatly influences its clinical application success. In this review, we have outlined various strategies used for DSF-based cancer therapy delivery, with most of these systems undergoing rigorous preclinical animal studies.

Optimizing the physicochemical properties of nanoparticles such as particle size, zeta potential, and surface functionalization is essential for optimal antitumor efficacy to minimize systemic toxicity and maximize delivery to the tumor. Furthermore, the use of novel tumor-targeting molecules like antibodies, aptamers, and peptides can reinforce specificity. Targeting biological barriers like cancer-associated fibrosis cells and extracellular matrices is also necessary for effective drug delivery into tumors.

Many delivery strategies for DSF-based cancer therapies have been developed, and these offer great potential in preclinical studies. Nevertheless, extensive evaluation to characterize safety and antitumor efficacy is required for clinical translation. The utilization of excipients already approved by the FDA or on the GRAS list can minimize regulatory obstacles and shorten R&D time, even though implementing new materials can offer more benefits.

For successful product development and commercialization, regulatory and marketing issues must be addressed. Strategies for building intellectual properties, proper regulatory compliance, and developing a scalable production process are essential. The lack of patent protection for the DSF drug molecule may discourage pharma companies from investing in clinical trials for DSF-based cancer therapies.

Therefore, designing novel formulations to improve antitumor efficacy while contributing to the development of intellectual properties is critical. In conclusion, DSF-based cancer therapy provides a promising avenue for antitumor treatment, with novel formulations and the use of tumor-targeting molecules current promising strategies. Proper evaluation, regulatory compliance, and intellectual property development are critical for successful clinical translation and commercialization of DSF-based cancer therapy.

## Conclusions

Recent studies have demonstrated that DSF is safe, efficient, cost-effective, and has great promise as an antitumor drug. Many first-line conventional antitumor drugs can be enhanced synergistically by DSF, thus providing new opportunities for the development of effective combination therapies. The current mechanisms of DSF’s antitumor effects are as follows. First, Inhibition of ROS scavenging pathways in cancer cells leads to cellular damage due to their massive accumulation. Second, regulation of NF-κB and JNK signaling pathway, which affect cell apoptosis. Third, regulation of intracellular protein degradation by promoting coagulation and binding of NPL4 protein, thereby acting as a degradation agent for p97 protein. Forth, effective induction of tumor cell growth inhibition and apoptosis by blocking the cell cycle process. Fifth, DSF can regulate the secretion and release of cytokines. Sixth, induces the ICD signaling pathway and stimulates T cells to attack tumor cells.

Surprisingly, DSF has been found to be able to modulate the anti-inflammatory and antitumor effects of immune cells and has great potential to become an effective immunomodulatory drug. In the area of adaptive immune response, DSF directly activates LCK-mediated TCR signaling to induce strong antitumor immunity. DSF/Cu^2+^ could restrain GSK3β activity by inhibiting PARP1, leading to the upregulation of PD-L1 expression. Combination therapy with DSF/Cu^2+^ and an anti-PD-1 antibody showed much better antitumor efficacy than monotherapy. In terms of cytokine/chemokines release, DSF could reduce expression of inflammatory cytokines and chemokines and reduced CD86 and MHC class II expressions during inflammation, autoimmune diseases, or tumors. In terms of ICD, DSF can up-regulate the expression of CRT protein to recruit effector T cells to attack tumor cells and exploit the immunogenic effect. These clues provide novel molecular insights into the therapeutic effect of DSF on cancers.

DSF is more effective and less costly and toxic, but it also has disadvantages. DSF has poor water solubility and is unstable in the body. For example, DSF degrades rapidly in an acidic gastric environment, and the active ingredients of the antitumor agent can easily fail. In addition, DSF has a plasma half-life of less than 4 min and is metabolized rapidly in the body, which makes the time for DSF to work effectively very short. In order to overcome the hydrophobic disadvantages of DSF, important progress has been made in the application of nano-transport systems. Nanoparticles loaded with DSF have been synthesized using polysaccharides, polyacrylates, PLGA and other macromolecular substances as carriers, showing advantages such as slow release and targeting. However, there are still limitations in the selection of materials and system construction of nanoparticle-loaded DSF systems. There are also limitations in the scope of research on the use of current delivery systems for tumor cells. Further research is needed on how to better and more selectively address the hydrophobicity of DSF. The combination of DSF, which reduces the protumor activity of macrophages, can significantly increase the number of cytotoxic CD8^+^ T cells in tumor cells when combined with PD-1 antibodies, enhancing the antitumor immune response and synergistically inhibiting the growth and metastasis of tumor cells.

In summary, this review has provided an overview of DSF-based cancer therapy, including its molecular structure, pharmacokinetics, signaling pathways, and mechanisms of action. Furthermore, we have focused on the immunomodulatory effects of DSF and discussed the emerging delivery strategies that could overcome the challenges associated with DSF-based antitumor therapies. While these delivery strategies show promise, rigorous evaluation and further studies are necessary to determine the safety and efficacy of DSF-based cancer therapy for clinical translation. With continued research and development, DSF-based cancer therapy holds great potential as an antitumor agent that could improve the outcomes of cancer patients and enhance the overall success rate of cancer therapy.

## Data Availability

The datasets generated and analyzed during the current study are available from the corresponding authors on reasonable request.
